# Repurposing Drugs for Synergistic Combination Therapies to Counteract Monkeypox Virus Tecovirimat Resistance

**DOI:** 10.3390/v17010092

**Published:** 2025-01-13

**Authors:** Haydar Witwit, Beatrice Cubitt, Roaa Khafaji, Esteban M. Castro, Miguel Goicoechea, Maria M. Lorenzo, Rafael Blasco, Luis Martinez-Sobrido, Juan C. de la Torre

**Affiliations:** 1 Department of Immunology and Microbiology, The Scripps Research Institute, La Jolla, CA 92037, USA; 2 Texas Biomedical Research Institute, San Antonio, TX 78227, USA; 3 Division of Infectious Diseases, Scripps Health, San Diego, CA 92103, USA; 4 Departamento de Biotecnología, INIA CSIC, 28040 Madrid, Spain

**Keywords:** monkeypox, antiviral, mpox, drug synergistic interactions, *Poxviridae*, VACV, vaccinia virus, drug repurposing, myristoylation, mycophenolate mofetil, IMP-1088, TPOXX, tecovirimat

## Abstract

The ongoing monkeypox (mpox) disease outbreak has spread to multiple countries in Central Africa and evidence indicates it is driven by a more virulent clade I monkeypox virus (MPXV) strain than the clade II strain associated with the 2022 global mpox outbreak, which led the WHO to declare this mpox outbreak a public health emergency of international concern. The FDA-approved small molecule antiviral tecovirimat (TPOXX) is recommended to treat mpox cases with severe symptoms, but the limited efficacy of TPOXX and the emergence of TPOXX resistant MPXV variants has challenged this medical practice of care and highlighted the urgent need for alternative therapeutic strategies. In this study we have used vaccinia virus (VACV) as a surrogate of MPXV to assess the antiviral efficacy of combination therapy of TPOXX together with mycophenolate mofetil (MMF), an FDA-approved immunosuppressive agent that we have shown to inhibit VACV and MPXV, or the N-myristoyltransferase (NMT) inhibitor IMP-1088. Both MMF and IMP-1088 drugs exhibited strong dose-dependent antiviral activity against VACV and mpox, and potent synergistic effects in conjunction with TPOXX. Our findings support combination therapy of direct-acting (TPOXX) and host-targeted (MMF and IMP-1088) antivirals as a promising approach to treat mpox and prevent the emergence and spread of TPOXX-resistant MPXV variants.

## 1. Introduction

Historically, MOPXV infections in humans have been rarely reported outside its endemic regions in Africa, but in 2022 a large number of monkeypox cases, caused by MPXV, were reported worldwide with sustained person-to-person transmission raising global health concerns [1]. There are two known distinct genetic MPXV clades: clade I is predominant in the Central Africa and can exhibit ~10% lethality in humans, whereas clade II is predominant in West Africa and associated with low virulence and lethality in humans, and the clade IIb lineage was responsible for the 2022 mpox global epidemic [2]. The limited antiviral drug pharmacopeia together with the rapid selection of drug-resistant viruses present a significant challenge to the medical community [3,4,5,6,7]. The recent outbreak of mpox in Central Africa illustrates this issue as TPOXX, a FDA-approved antiviral for the treatment of smallpox and under the CDC-held Expanded Access-Investigational New Drug (EA-IND) protocol certain mpox patients, failed to improve outcomes in individuals with clade I MPXV infection in a recent clinical trial in the Democratic Republic of the Congo (DRC) [8,9,10]. This lack of efficacy may have been due to sub-optimal potency and de novo drug resistance as TPOXX resistant MPXV variants are readily found in clinical cases of mpox, especially in immunosuppressed patients on long-term treatment [11]. MPXV is classified within the *Orthopoxvirus* genus, which includes smallpox and vaccinia virus (VACV), in the family *Poxviridae*. Members of this genus possess linear double-stranded DNA (dsDNA) genomes and a life cycle restricted to the cytoplasm of infected cells [12], involving four distinct infectious virus stages: the intracellular mature virion (IMV), the intracellular enveloped virion (IEV), the cell-associated enveloped virion (CEV), and the extracellular enveloped virion (EEV) [13]. The small molecule antiviral TPOXX targets the viral F13 protein resulting in inhibition of IMV wrapping in both VACV and MPXV [14,15]. The protein encoded by F13L, a conserved gene within the *Poxviridae* family, is essential for virus envelopment and release [14,16], and likely for efficient viral entry [17,18]. VACV can be used as an accurate surrogate for the investigation of MPXV antivirals, as both share functionally conserved proteins in their genomes [19,20]. Both viruses exhibit similar resistance mechanisms to TPOXX associated with mutations in the F13L gene. VACV resistance to TPOXX has been linked to several mutations including G277C in the F13L gene [21], whereas T289A and A290V mutations in F13L have been confirmed to confer MPXV with resistance to TPOXX [11]. Several additional mutations including R291K, S215F, and P243S in the F13L gene have been linked, but yet to be confirmed, to MPXV resistance to TPOXX [11]. MPXV mutations associated with increased resistance to TPOXX have been identified among severely immunocompromised mpox patients who required prolonged courses of TPOXX [11,22,23]. However, spread of TPOXX-resistant MPXV has been also reported among persons with no previous TPOXX treatment [22]. The genome of MPXV Clade Ib contains large deletions in gene OPG032 [24,25], but these deletions do not contribute to TPOXX resistance. Likewise, none of the multiple mutations consistent with APOBEC3-mediated cytosine deamination present in the genome of Clade Ib MPXV has been directly implicated in TPOXX resistance [11,24]. However, the recent outbreak of clade Ib MPXV [24] in DRC [25] and Sweden [26,27], together with the recently released results of the international clinical trial STOMP (NCT05597735), highlight the challenges posed by the limited efficacy of TPOXX against both clades of MPXV, and the urgent need for alternative antivirals [9,10].

We have documented that mycophenolate mofetil (MMF), an FDA-approved drug, has potent antiviral activity against both VACV and MPXV in cell-based assays [20]. N-myristoylation has been shown to be crucial for the propagation of several viruses [28,29], and VACV multiplication was effectively inhibited by the NMT inhibitor IMP-1088 [30]. In this study, we used VACV as a surrogate model for MPXV to assess the efficacy of combination therapy based on the combination of direct-acting (TPOXX) and host-targeting (MMF or IMP-1088) antivirals as a strategy to counteract MPXV TPOXX resistance. This approach targets multiple steps of the virus life cycle, which via synergistic effects can result in more effective control of viral burden and associated improved clinical outcomes of mpox, as well as posing a high genetic barrier to the emergence of drug-resistant MPXV.

## 2. Materials and Methods

### 2.1. Cell Lines and Viruses

Homo sapiens A549 (ATCC CCL-185) cell line was maintained in Dulbecco’s modified Eagle medium (DMEM) (ThermoFisher Scientific, Waltham, MA, USA) containing 10% heat-inactivated fetal bovine serum (FBS), 2 mM L-glutamine, 100 μg/mL streptomycin, and 100 U/mL penicillin. The bi-reporter recombinant VACV expressing fluorescent protein (GFP, downstream of the A27L locus) and nanoluciferase (Nluc, downstream of the F13L locus), rVACV Nluc/GFP, was previously described and characterized [31,32]. MPXV 2003 (NR-2500) was obtained from BEI Resources (USA).

### 2.2. Compounds

Compounds in the ReFRAME library with antiviral activity against RNA have been described [20,33], and were purchased from MedChem Exp (USA) and include mycophenolate mofetil, MMF (cat. No. HY-B0199), buparvaquone (cat. No. HY-17581), OSU-03012 (cat. No. HY-10547), AVN-944 (cat. No. HY-13560), brequinar (cat. No. HY-108325), Valinomycin (cat. No. HY-N6693). The NMT inhibitor IMP-1088 was purchased from Cayman Chemical (USA) (cat. No. 25366-1) and DDD85646 (Synonyms: IMP-366) was purchased from MedChem Exp (Cat. No.: HY-103056). Tecovirimat (Synonyms: ST-246), referred to as TPOXX here, was purchased from MedChem Exp (Cat. No.: HY- HY-14805).

### 2.3. Determination of Compound EC_50_

A549 cells were seeded on 96-well clear-bottom black plates (4.0 × 10^4^ cells/well) and infected (MOI 0.01) 20 h later with rVACV Nluc/GFP using four technical replicates. After a 60 min adsorption, the virus inoculum was aspirated off and compound-containing media was added to the cells. At 48 h pi, the cells were fixed with 4% paraformaldehyde (PFA) for 30 min, then washed once with PBS, and GFP expression was determined by fluorescence using a fluorescent plate reader BioTek Cytation 5 (Agilent, Santa Clara, CA, USA). The mean relative fluorescence units were normalized to vehicle control (DMSO)-treated cells, which were assigned a value of 100%. The half-maximal effective concentrations (EC_50_) were determined using GraphPad Prism (GraphPad Software, Boston, MA USA), v10 (Prism10).

### 2.4. Determination of Compound CC_50_

Cell viability was determined using the 4′,6-diamidino-2-phenylindole (DAPI) staining, which is a blue-fluorescent DNA stain that exhibits about 20-fold enhancement of fluorescence upon binding to AT regions of double-stranded DNA. A549 cells were plated on 96-well clear-bottom plates (4.0 × 10^4^ cells/well) using four technical replicates. Serial dilutions of each compound were added to the cells, and at 48 h after the drug treatment, cells were fixed with 8% PFA (final 4%) and incubated for 30 min, then washed once with DPBS; signals were quantified using the BioTek Cytation 5. The resulting optical densities were normalized to the vehicle-treated (DMSO) control samples, which were assigned a value of 100%. The half-maximal cytotoxic concentrations (CC_50_) were determined using Prism10.

### 2.5. Drug Combination Assays in VACV-Infected Cells

A549 cells were seeded on 96-well clear-bottom black plates (4.0 × 10^4^ cells/well) and infected (MOI 0.01) 20 h later with rVACV Nluc/GFP. After a 60 min adsorption, the virus inoculum was aspirated, and compound-containing media were added to the cells. The compounds were prepared in serial dilution, 2× concentration, then added to predetermined matrix of compounds mixture, to bring the final concentration to 1×. At 48 h pi, cell culture supernatants (CCSs) were collected, then cells were fixed with 4% paraformaldehyde, and GFP expression levels were determined using a fluorescent plate reader (BioTek Cytation 5). Mean relative fluorescence units were normalized to vehicle-treated (DMSO) control cells, which were assigned a value of 100%. Compounds’ half-maximal effective concentrations (EC_50_) were determined using Prism10. To assess the effect of drug combination treatments in production of rVACV Nluc/GFP infectious progeny, CCSs were diluted (1:10) and 50 µL used to infect A549 cells seeded on 96-well clear-bottom black plates (4.0 × 10^4^ cells/well). After a 60 min adsorption, the virus inoculum was aspirated, and media were added to the cells. At 48 h pi, CCSs were collected, and cells fixed with 4% paraformaldehyde, and GFP expression levels determined using a fluorescent plate reader (BioTek Cytation 5). Mean relative fluorescence units were normalized to vehicle control (DMSO)-treated cells, which were assigned a value of 100%. The half-maximal effective concentrations (EC_50_) were determined using Prism10.

For synergetic studies, we prepared an 8-by-8 matrix (i.e., two drugs were tested for all possible 64 combinations of the two drugs). A549 cells, 4 × 10^4^/well, were seeded in 96-well, black, clear-bottom plates in a final volume of 100 µL/well. The plates were incubated at 37 °C with 5% CO_2_ overnight. Stock solutions of each drug (A and B) were made at 2× in 3000 µL. U-bottom 96-well plates with 125 µL of media were prepared, except first row or column, then 2× drug stock was added into first row or column, and 1:2 serial dilutions were performed. Samples in the 2× plates were mixed into a 1:1 mixture to obtain 1× final concentration ready to be added to wells of the plate containing infected cells after a 60 min adsorption period (SF1). Normalized values from matrix data were analyzed using SynergyFinder+ software (https://synergyfinder.org/), an open-access platform for multidrug combination synergies [34]. For the combination synergy model, we used four models: ZIP, HSA, Loewe, and Bliss; however, we sorted synergetic score based on ZIP’s logistic growth fit and scores because both ZIP and Loewe can interpolate from logistic growth fit, as opposed to HSA and Bliss where they align with linear fit. Also, the ZIP model assumed independent mechanistic pathways involved in the synergy. In the synergy matrix, a 100% value of GFP signal indicates a total inhibition, while 0% means no inhibition. The initial combination therapy assessment was carried out using one biological replicate, while the rest of the combination therapy assays were carried out using three independent biological replicates. Complete baseline correction was conducted on all values in SynergyFinder+.

### 2.6. Drug Combination Assays in MPXV-Infected Cells

A549 cells (4 × 10^4^ cells/96-well, triplicate) were infected with MPXV 2003 (BEI Resources NR-2500) (MOI 0.01–0.02, 50 µL/well in DMEM/2% FBS, 1% PSG) for 1 h at 37 °C with occasional shaking. After the adsorption time, viral inoculum was aspirated off and 100 µL of serial dilutions from combination plate TPOXX and MMF or IMP-1088 and MMF was added to cells. At 24 h pi, CCS was aspirated off and cells were fixed in 10% neutral buffered formalin at 4 °C. After 24 h, cells were washed with ddH2O 3× and overlayed with 50 µL of 1× DAPI in PBS for 30 min at room temperature (RT). Cells were washed with PBS and imaged for DAPI fluorescence using a Synergy H1 Hybrid Multi-Mode Reader (Agilent, Santa Clara, CA, USA). Cells were then washed and permeabilized with 0.5% Triton X-100 in 1× PBS for 15 min at RT, followed by 2 h incubation at 37 °C in PBS containing 2.5% BSA to block non-specific antibody binding. Cells were incubated with a rabbit polyclonal serum to VACV-A33R (BEI Resources NR-628) (1:5000) in PBS/2.5% BSA for 2 h at 37 °C. After washing, cells were incubated with a secondary anti-rabbit antibody, washed and incubated with ABC reagent for 45 min at 37 °C, followed by incubation with DAB reagent for 15 min at RT (VectorLabs ABC HRP and DAB). Cells were allowed to dry and read using the Bioreader 7000 Fz machine (BIOSYS, Miami, FL, USA). MPVX experiments were performed in BSL3 facilities at Texas Biomedical Research Institute.

## 3. Results

### 3.1. Dose–Response Inhibitory Effect of Selected Drugs on VACV Multiplication

**Figure 1:**
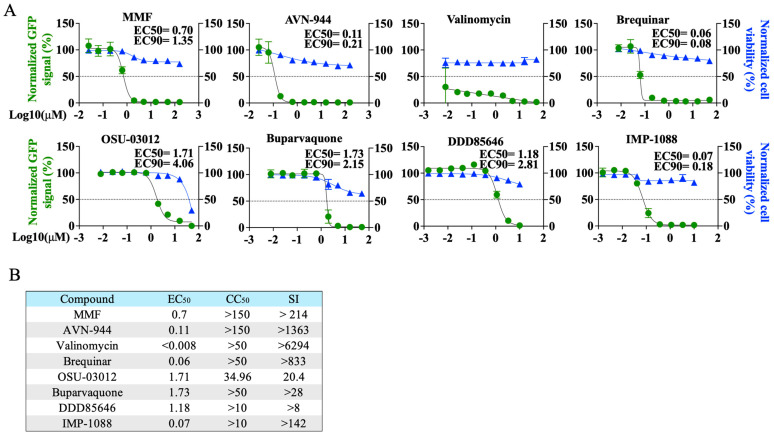
Dose–response of selected drugs in A549 cells infected with VACV. (**A**) A549 cells were seeded at 4 *×* 10^4^/well in optical 96 well plates, and 16 h later infected with rVACV Nluc/GFP at MOI of 0.01. After 60 min adsorption, the virus inoculum was aspirated and media containing serial dilutions (1:3) of the indicated drugs added to cells. At 48 h pi, cells were fixed and inhibition of rVACV Nluc/GFP replication was assessed by quantifying levels of GFP expression (green line). Cell viability was determined by DAPI staining (blue line) using a BioTek Cytation 5 reader. The EC_50_ and EC_90_ values for each compound were calculated using a sigmoidal dose–response four-parameter and log(agonist) *vs.* response model with constrain F value set to constant 10, respectively, with GraphPad Prism. The horizontal dotted lines indicate 50% inhibition. Data are means and SDs of viral inhibition from quadruplicate wells (n  =  4). (**B**) EC_50_, CC_50_, and selectivity index (SI) of each compound.

To select compounds for combination therapy with TPOXX, we conducted dose–response assays to confirm the previously shown anti-VACV activity of MMF, AVN-944, brequinar, valinomycin, OSU-03012, and buparvaquone [20]. In addition, we examined the dose response of the NMT inhibitors DDD85646 and IMP-1088 on VACV multiplication. All eight tested compounds exhibited potent dose-dependent inhibitory effect on VACV multiplication at 48 h pi (Figure 1A) and had robust selectivity indices (SIs) (Figure 1B), similar to described findings that were measured at 24 h pi [20].

MMF is a prodrug that serves as a potent irreversible inhibitor of inosine-*5′*-monophosphate dehydrogenase (IMPDH), leading to the depletion of guanosine nucleotides; it is selective for isoform II of IMPDH [35]. MMF has been shown to exhibit antiviral activity against different viruses [36,37,38], including VACV [39,40]. AVN-944 is a specific and potent pan-inhibitor of IMPDH [41]. Brequinar, an inhibitor of dihydroorotate dehydrogenase (DHODH), has demonstrated potential in synergistic studies [42]. Valinomycin acts as an ionophore, disrupting the potassium ion gradient across the cell membrane [43]. OSU-03012 inhibits phosphoinositide-dependent kinase 1, thereby interfering with Akt signaling, with a preference for the GRP78 chaperone [44,45,46]. Buparvaquone disrupts cytochrome b in eukaryotic cells [47,48]. Both DDD85646 and its analog IMP-1088 are highly selective pan-NMT inhibitors known to exhibit antiviral activity against viruses, including VACV, which rely on myristoylated proteins to complete their life cycle [28,29,30]. Importantly, the IMP-1088 derivative NMT inhibitor PCLX-001 has advanced to a phase II clinical drug trial and has exhibited a good safety profile in phase I, where it completed six dose escalations without dose-limiting toxicities, reaching clinically active exposure levels [49,50,51].

### 3.2. Assessment of Synergistic Anti-VACV Activity of Selected Drugs in Combination with TPOXX

**Figure 2:**
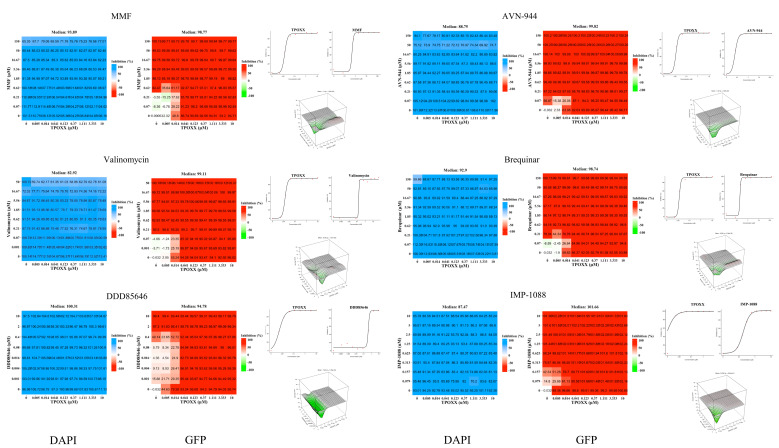
Synergistic effects of selected drugs in combination with TPOXX. A549 cells were seeded at 4 *×* 10^4^/well in optical 96-well plates, and 16 h later infected with rVACV Nluc/GFP at MOI of 0.01. After 60 min adsorption, the virus inoculum was removed and medium containing TPOXX or the indicated drug combinations and concentrations added to cells, and infection allowed to proceed. At 48 h post-infection (h pi), inhibition of rVACV Nluc/GFP multiplication was assessed based on levels of GFP expression and cell viability determined by DAPI staining using the BioTek Cytation 5 plate reader. The heat maps of synergy matrices, dose–response, and three-dimensional synergy plot and scores were determined using SynergyFinder+ software, the table template was used to input the data, then the data were uploaded to the software. In the specify response step of the software, the % inhibition option for GFP signal or % viability option for DAPI was used, respectively.

To investigate the synergistic activity of the different compounds with TPOXX, we designed a matrix to screen for synergy using a 96-well plate format. All six tested compounds showed strong anti-VACV activity in combination therapy with TPOXX (Figure 2). We measured their synergistic effect with TPOXX based on four models: ZIP, Bliss, Loewe, and HSA; only the three-dimensional plot of ZIP synergy depiction is shown. All tested combinations demonstrated significant inhibition. Nevertheless, the high dose applied in this experiment exceeded the detection threshold of the synergy software, preventing us from obtaining accurate measurements of the synergistic activity. Based on the compounds known features, including their clinical profiles, we selected MMF and IMP-1088 for further detailed investigation of their use in combination therapy with TPOXX.

### 3.3. Detailed Assessment of the Synergistic Anti-VACV Activity of MMF and IMP-1088 in Combination with TPOXX

**Figure 3:**
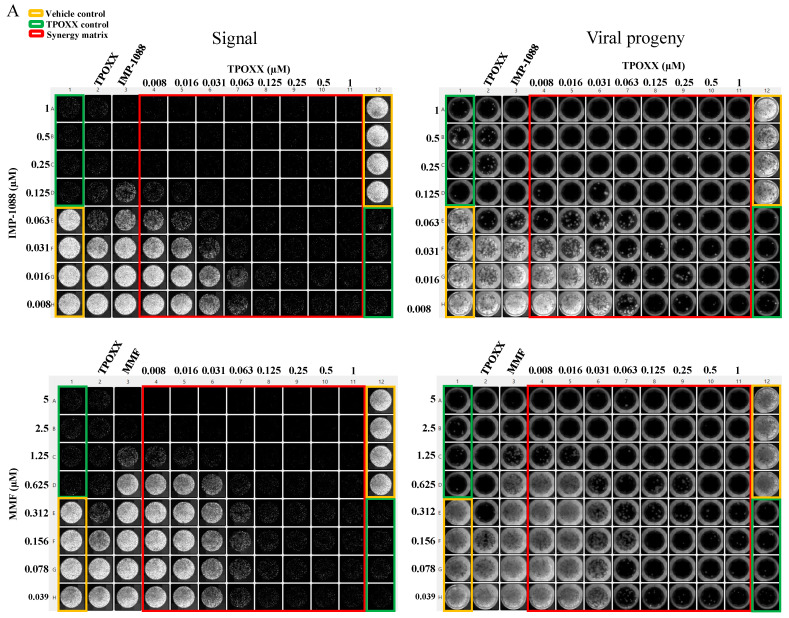

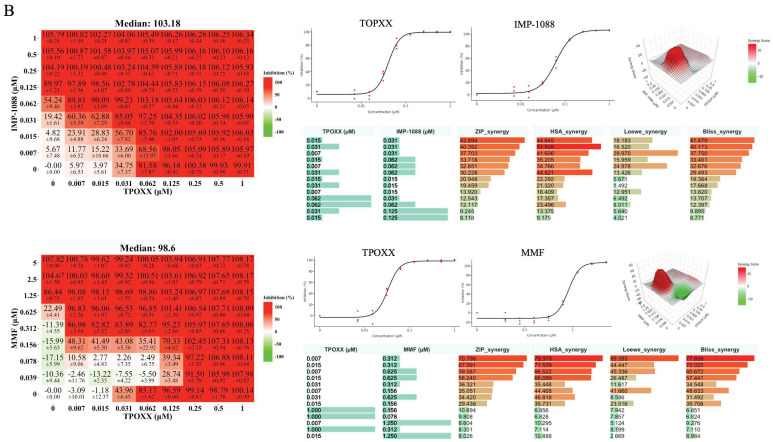
Synergistic anti-VACV activity of MMF or IMP-1088 in combination with TPOXX. A549 cells were seeded at 4 × 10^4^/well in optical 96-well plates, and 16 h later infected with rVACV Nluc/GFP at MOI of 0.01. After 60 min adsorption, the virus inoculum was removed and the indicated drug combinations and concentrations added to the cells. (**A**) At 48 h pi, inhibition of rVACV Nluc/GFP multiplication was assessed based on GFP expression levels using a Celigo machine. Screenshots of representative plate of the three biological replicates, referred to as Signal, were used to capture the plate results (left side panel). CCSs were collected, diluted into 1:10 (total of 150 µL) in DMEM containing 10% FBS and 50 µL used to infect fresh A549 cells (seeded at 4 × 10^4^/well in 96 clear well format). After 60 min adsorption, virus inoculum was removed and fresh DMEM containing 10% FBS (100 µL/96 well) added to cells. At 48 h pi, cells were fixed with 4% PFA and imaged with a Celigo machine (right panel); representative single plates of the three biological replicates are shown. (**B**) The heat maps of synergy matrices, dose–response, three-dimensional synergy plots and scores were obtained, using SynergyFinder+ software, for the three biological replicates. Bar plots of the synergistic dose–response were obtained using RStudio software (version2024.04.2 Build 764). The % inhibition option was set for GFP signals.

To test MMF and IMP-1088 synergistic effects with TPOXX, we treated VACV-infected cells with the indicated drug combinations, starting at 1 μM for TPOXX and IMP-1088, and 5 μM for MMF, and using two-fold serial dilutions. MMF and IMP-1088 showed strong dose–response synergistic effect in combination with TPOXX with ZIP synergy scores reaching values > 43 and 70 for IMP-1088 and MMF, respectively (Figure 3). To assess the effect of the combination therapy on production of extracellular infectious viral progeny (EEV), we collected the CCSs from the drug combination treatments and used them to infect fresh A549 cells. We found that combination therapy potently inhibited production of EEV progeny (Figure 3).

### 3.4. Effect of Combination Therapy on VACV Multi-Step Growth Kinetics

**Figure 4:**
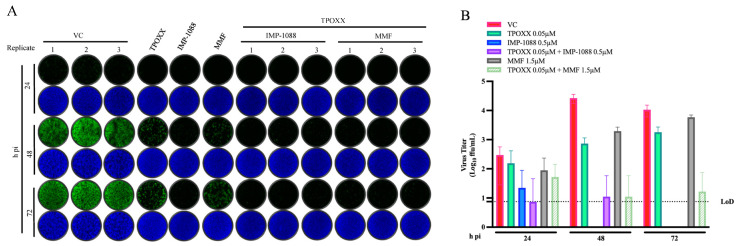
Effect of combination therapy of MMF or IMP-1088 with TPOXX on VACV multi-step growth kinetics. A549 cells were seeded at 1.25 *×* 10^5^/well in optical 24-well plates, and 16 h later infected with rVACV Nluc/GFP at MOI of 0.01. After 60 min adsorption, the virus inoculum was removed and media containing the indicated drugs and concentrations (0.05 µM TPOXX, 1.5 µM MMF and 0.5 µM IMP-1088 as single drugs or combinations of TPOXX and MMF or IMP-1088) were added to cells. At 48 h pi, CCSs were collected and cells fixed with 4% PFA. Images showing GFP expression were obtained using Keyence XZ-710 (**A**). Virus titers in CCS were determined by FFA using A549 cells in a 96-well plate format (**B**).

To assess the effect combination therapy on VACV growth kinetics during multiple rounds of infection, we infected A549 cells with rVACV Nluc/GFP at MOI of 0.01 and treated VACV-infected A549 cells with the indicated drug combinations. At the indicated h pi, we determined titers of infectious rVACV Nluc/GFP in CCS (Figure 4). Combination therapy of MMF or IMP-1088 with TPOXX resulted in strong inhibition of VACV multiplication (Figure 4A) and production of peak titers of infectious VACV at 72 h pi (Figure 4B). At 72 h pi, combination therapy of IMP-1088 + TPOXX resulted in titers of infectious VACV progeny below detection levels (>4 log reduction), whereas combination therapy of MMF + TPOXX resulted in barely detectable levels (>3 log reduction) of infectious VACV progeny compared with vehicle control-treated and VACV-infected samples.

### 3.5. Effect of IMP-1088 and MMF Combination Therapy on VACV Infection

**Figure 5:**
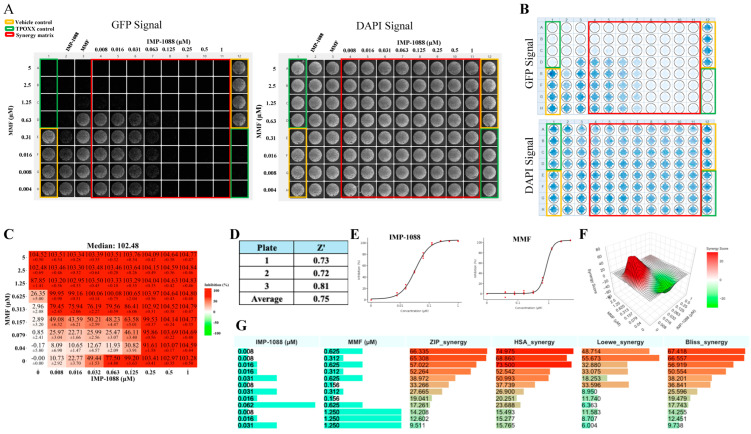
Synergistic antiviral activity of MMF and IMP-1088 combination treatment. A549 cells were seeded at 4 *×* 10^4^/well in 96 optical well format; next day, cells were infected with rVACV Nluc/GFP at MOI of 0.01 and adsorbed for 60 min, a 1:2 serial dilution of 2× of the indicated drugs were combined into 1x, then replaced the adsorption inocula and incubated at 37 °C. (**A**) Visual images of the effect of combination therapy on VACV propagation. At 48 h pi, inhibition of rVACV Nluc/GFP propagation among cells was assessed based on GFP expression (left side panel), and cell viability was assessed based on DAPI signal (right side panel). GFP and DAPI signals were obtained using a Celigo machine and used to generate screenshots of the plate results. (**B**) Total GFP and DAPI signals for each well were obtained using a BioTek Cytation 5. (**C**–**G**) Quantification of combination therapy inhibitory effect on VACV propagation. Inhibition of rVACV Nluc/GFP viral replication was assessed by quantifying fluorescent GFP and cell viability determined by DAPI staining using a BioTek Cytation 5. The heat maps of synergy matrices (**C**), dose–response (**E**), three-dimensional synergy plot and scores (**F**), and bar plot of the synergetic dose–response (**G**) were obtained using SynergyFinder+ software or its R code using RStudio software (version2024.04.2 Build 764); the inhibition setting was used for GFP signals. The Z’ scores (**D**) were calculated using the following equation: Z’ = 1-(3(standard deviation of vehicle control-standard deviation of negative control))/(average of vehicle control-average of negative control). These experiments were carried out in three biological replicates with Z’ averaged at 0.75 (three biological replicates with 24 technical replicates for vehicle control and negative control).

To determine whether IMP-1088 and MMF exhibit synergistic antiviral activity against VACV, we treated VACV-infected cells with the indicated combinations of IMP-1088 and MMF. We found that IMP-1088 and MMF exhibited a strong dose-dependent synergistic response with ZIP synergy score values > 66 (Figure 5). Importantly, the combination of MMF and IMP-1088 at drug concentrations associated with high synergy score values lacked noticeable effects on cell viability (Table S1).

### 3.6. Effect of Combination Therapy on MPXV Infection

**Figure 6:**
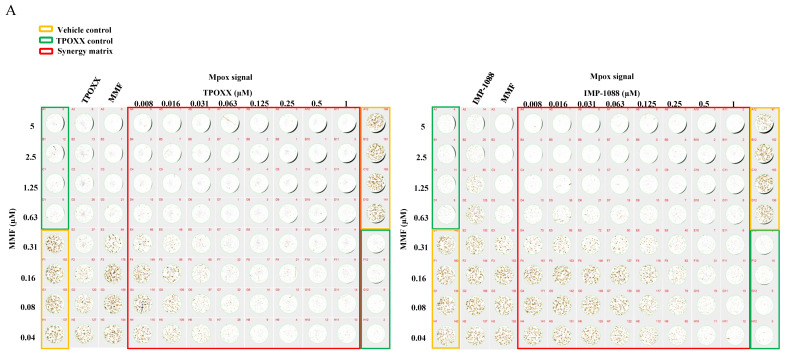

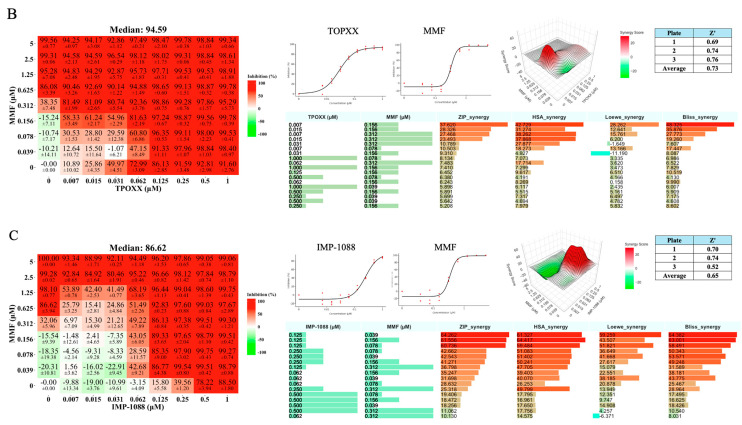
Combination treatment against MPXV. A549 cells were seeded at 4 × 10^4^/well in 96 optical well format. Next day, cells were infected with MPXV at MOI 0.01–0.02 and adsorbed for 60 min, a 2× of 1:2 serial dilution of the indicated drugs was combined into 1×, then replaced the adsorption inocula and incubated at 37 °C. (**A**) At 48 h pi, inhibition of MPXV replication was assessed by IF imaging using a Bioreader 7000 Fz. Representative plates of the three biological replicates are shown. (**B**,**C**) Quantification of the effect of combination therapy on MPXV propagation. Heat maps of synergy matrices, dose response of monotherapy, three-dimensional ZIP synergy plot, Z score, and bar plot of the scores of four synergistic models for TPOXX and MMF (**B**), and IMP-1088 and MMF (**C**) combination therapy protocols are shown. Bar plots of the synergetic dose response were obtained using SynergyFinder+ software or its R code using RStudio software (version2024.04.2 Build 764). The inhibition setting was selected for IF signals. The Z’ score were calculated using the following equation: Z’ = 1-(3(standard deviation of vehicle control-standard deviation of negative control))/(average of vehicle control-average of negative control). These experiments were carried out in three biological replicates with Z’ averaged 0.73 (for TPOXX and MMF) and 0.65 (for IMP-1088 and MMF) (three biological replicates with 24 technical replicates for vehicle control and negative control).

We next examined whether our findings on the efficacy of antiviral combination therapy to inhibit VACV multiplication in cultured cells were also applicable to infection with bona fide MPXV. Combination therapy of TPOXX and MMF exhibited a strong dose-dependent synergistic antiviral activity against MPXV, with ZIP synergy score values > 37 (Figure 6A,B). Likewise, combination therapy of IMP-1088 and MMF also exhibited a strong dose-dependent synergistic response with ZIP synergy score values > 64 (Figure 6A,C).

### 3.7. Triple Therapy in VACV

**Figure 7:**
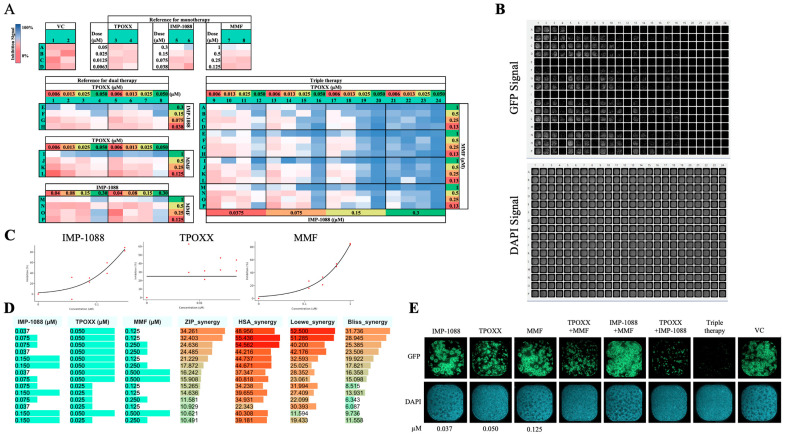
Effect of triple combination therapy of TPOXX, MMF, and IMP-1088 on VACV multiplication. A549 cells were seeded at 4 *×* 10^4^/well in optical 96-well plates, and 16 h later infected with rVACV Nluc/GFP at MOI of 0.01. After 60 min adsorption, the virus inoculum was removed and the indicated drug combinations and concentrations added to the cells. At 48 h pi, inhibition of rVACV Nluc/GFP multiplication was assessed based on GFP expression levels (green signal) using a BioTek Cytation 5. (**A**) Heatmap results and plate matrix assignment. Reference vehicle control (VC) was assigned to columns 1 and 2 (rows 1–4), the dose–response with four, 1:2, serial dilutions, was assigned to columns 3 and 4 for TPOXX starting at 0.05 µM, columns 5 and 6 for IMP-1088 at 0.3 µM, and columns 7 and 8 for MMF at 1 µM. The blocks for dual therapy were assigned to columns 1–4 and 5–8 for two replicates, where rows E-H were assigned for TPOXX and IMP-1088, rows I-L for TPOXX and MMF, and rows M-P for IMP-1088 and MMF. Columns 9–24 were segregated into 4 × 4 blocks, and assigned the TPOXX and MMF dual therapy, then IMP-1088 was added at the indicated doses, starting with highest dose from right to left. (**B**) IF images of GFP (top) and DAPI (bottom) signal of the plate. (**C**) Dose–response of monotherapy for IMP-1088, TPOXX, and MMF plotted using SynergyFinder+ software. (**D**) Synergistic models showing dose–response synergistic effect as calculated by SynergyFinder+ software. (**E**) Fluorescent and DAPI examples of mono-, dual-, and triple therapy of IMP-1088 (0.037 µM), TPOXX (0.05 µM), and MMF (0.125 µM). Images were obtained with a Celigo machine at 4 µ pixel resolution.

To explore whether triple combination therapy could result in a stronger inhibition at lower doses of each drug, we designed an experiment to investigate the synergistic effect of IMP-1088, TPOXX, and MMF at doses starting at 0.3 µM, 0.05 µM, and 1 µM, respectively, and serially diluted (1:2). We observed a strong dose–response synergistic effect among the three drugs reaching over 34, 48, 52, and 31 scores of the ZIP, HSA, Loewe, and Bliss models, respectively (Figure 7). This strong synergistic antiviral effect occurred in the absence of noticeable effects on cell viability (Figure 7B). Consistent with its potent antiviral activity, this triple combination therapy protected A549 cells from MPXV-induced cytopathic effect (Figure 7E).

## 4. Discussion

The mpox disease continues to spread in Africa and other parts of the world, posing a threat to global health security. This situation is exacerbated by the emergence of more recent virulent forms of MPXV, limited orthopoxvirus vaccines repurposed for prevention of mpox, the very narrow arsenal of antivirals effective against MPXV, and the emergence of viral variants with increased resistance to TPOXX, currently the only drug available for use to treat mpox. Hence, the development of novel therapeutics to treat mpox represents an important unmet clinical need. In this study, we have presented evidence supporting the use of two safe drugs, MMF and the NMT inhibitor IMP-1088, either alone or in combination with TPOXX, as potential therapeutic strategies against MPXV. We [20] and others [52] have shown that MMF inhibits multiplication of VACV and MPXV, and the use of MMF as an antiviral has been successful in treating other viral infections [36,37]. Evidence indicates that this antiviral activity of MMF is mediated by the ability of its metabolite mycophenolic acid to inhibit IMPDH, a key enzyme in purine biosynthesis and DNA replication [35]. MMF is an immunosuppressant that is indicated for transplant recipients as prophylactic therapy [53]; however, other indications in dermatological and rheumatological disease are well established [54,55]. The immunosuppressive effect of MMF is caused by its cytostatic effect on lymphocytes due to prolonged use, which is related to the high dependence of activated lymphocytes on robust IMPDH activity to generate the high purine levels required for active lymphocyte proliferation. Accordingly, no differences in lymphocyte cell count were observed between MMF-treated and control groups at day 3 post-treatment [56,57], which provides a therapeutic window for the antiviral use of MMF with limited cytostatic effect on lymphocytes. The use of MMF in combination therapy allows effective lower doses of MMF, and short treatment duration, which will further contribute to minimize MMF immunosuppressive activity while maintaining its potent antiviral activity. Using the myristoylator bioinformatic tool that predicts potential myristoylation signals in proteins [58,59], we identified four proteins in VACV and MPXV containing predicted myristoylation sites, including MPXVgp079, the counterpart of VACV G9R; MPXVgp080, the IMV membrane protein homologous to VACV L1R; MPXVgp127, the counterpart of VACV A16L. However, in contrast to the strong myristoylation signal exhibited by VACV E7R, its MPXV counterpart gp055, also known as F6R, is not predicted to be myristoylated. Likewise, MPXVgp039, but not its VACV counterpart F7L, shows probability of myristoylation. (Table S2). The findings from our bioinformatics analysis are consistent with published data on acylation of VACV proteins [60]. G9R [30,61], A16L [62], and L1R [63,64] proteins are components of the VACV entry-fusion complex and essential for virus multiplication, and the NMT inhibitor IMP-1088 potently inhibited VACV multiplication via targeting mainly myristoylation of the L1R protein [30,61]. Multiplication of VACV may require myristoylation of specific host cell proteins, and IMP-1088-mediated inhibition of this process may contribute to the anti-VACV activity of IMP-1088. The PCLX-001 NMT inhibitor has successfully completed phase I clinical trial and advanced toward phase II trial [49,50,51], supporting the safety of NMT inhibitors.

Combination therapy of antivirals with synergistic effects can pose a high genetic barrier to the emergence of drug-resistant viral variants that often jeopardize monotherapy approaches, as well as facilitate the use of reduced drug doses within therapeutic range and alleviate, or prevent, side effects associated with high drug doses used in monotherapy. Combination antiviral therapy can also help to counteract the ability of many viruses, including orthopoxviruses [65,66,67], to evade host cell antiviral pathways such as interferon (IFN), RNAse L, and PKR [68,69,70].

The success of combination therapy is illustrated by the current therapeutic approaches to treat HIV and HCV infections involving combinations of two to four antivirals targeting different steps of the virus life cycle [71,72,73,74,75], which promotes synergistic antiviral effects [76,77] that raise a genetic barrier to the emergence of drug-resistant viruses [78,79]. This situation is particularly relevant to MPXV infections of immunocompromised individuals, where prolonged virus multiplication can favor selection and human-to-human propagation of drug-resistant MPXV variants.

## Data Availability

Data are contained within the article and supplementary materials.

## Supplementary Materials

The following supporting information can be downloaded at: https://www.mdpi.com/article/10.3390/v17010092/s1. Figure S1. Schematic representation of experimental design for the synergistic studies. Table S1. Dose response synergistic score for IMP-1088 and MMF along with their cell viabilities for the corresponding doses. Table S2. Prediction of myristoylated proteins in MPXV or VACV shows scores, predicted targeted sequence for myristoylation and visual depiction using both Myristoylator and MYR predictor tools.
