# Supplementary material for: Repurposing Drugs for Synergistic Combination Therapies to Counteract Monkeypox Virus Tecovirimat Resistance
Source: Viruses. 2025 Jan 13;17(1):92. doi: 10.3390/v17010092 (PMC11769280; doi:10.3390/v17010092)
Supplement: Supplementary file 1 [file viruses-17-00092-s001.zip › viruses-3413041-supplementary.pdf]

viruses

Article

Repurposing Drugs for Synergistic Combination Therapies to Counteract Monkeypox Virus Tecovirimat Resistance

Haydar Witwit [1], Beatrice Cubitt [1], Roaa Khafaji [1], Esteban M. Castro [2], Miguel Goicoechea [3], Maria M. Lorenzo [4], Rafael Blasco [4], Luis Martinez-Sobrido [2] and Juan C. de la Torre [1,*]

[1] Department of Immunology and Microbiology, The Scripps Research Institute, La Jolla, CA 92037, USA
[2] Texas Biomedical Research Institute, San Antonio, TX, USA
[3] Division of Infectious Diseases, Scripps Health, San Diego, CA, USA
[4] Departamento de Biotecnología, INIA CSIC, Madrid, Spain
* Correspondence: juanct@scripps.edu

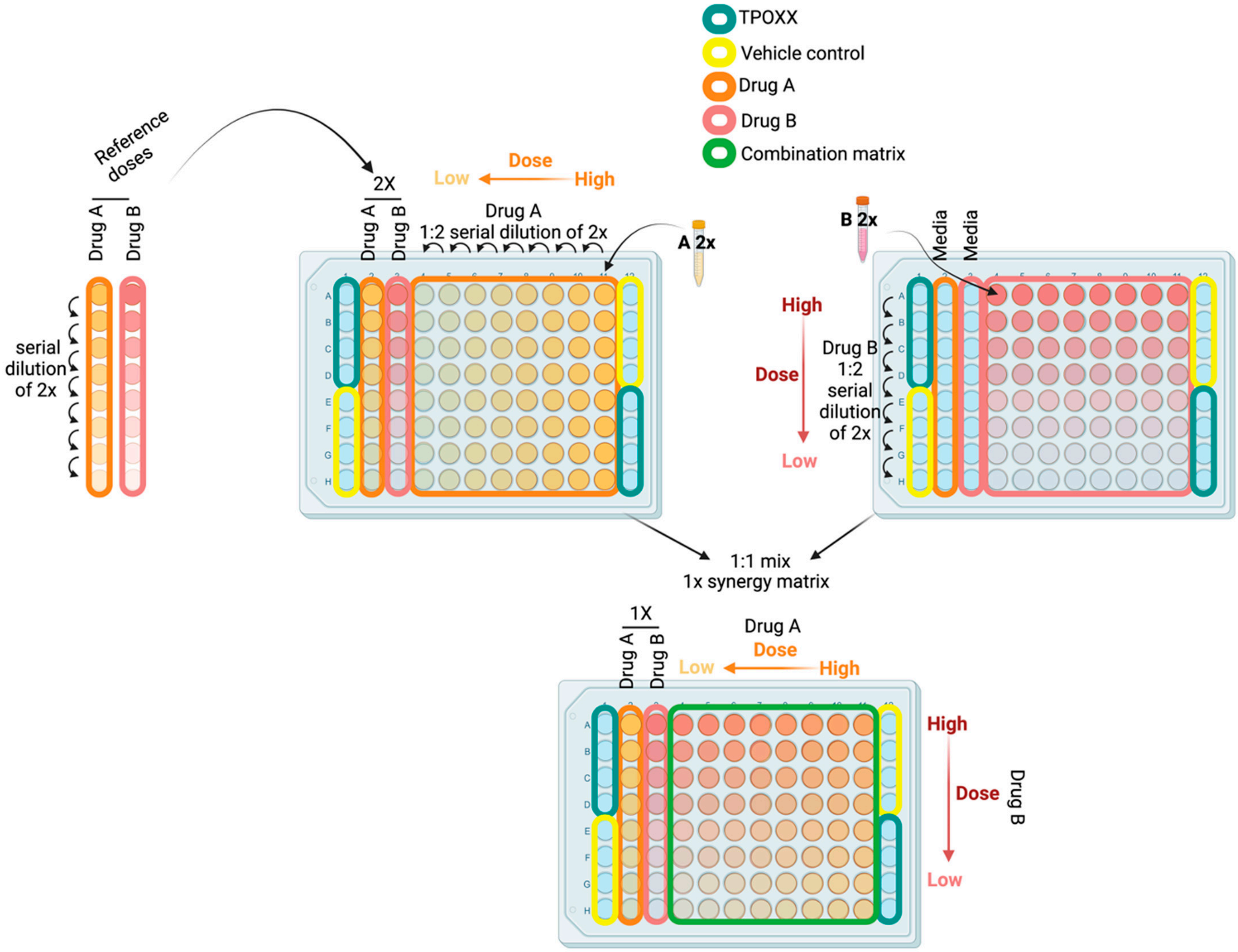

Figure S1. Schematic representation of experimental design for the synergistic studies. The experimental setting uses three 96-well plates. **Plate A:** 1) Columns 2 and 3 contain serial dilutions (1:2) of reference doses (2x concentration) for drugs A and B, respectively. 2) Column 11 wells contain drug A at 2x concentration that are serially diluted from right to left of the plate (column 11 towards column 4). 3) Wells 1A-1D and 12E-12H contain TPOXX, whereas wells 1E-1H and 12A-12D contain vehicle control (VC). **Plate B:** 1) Columns 2 and 3 contain media. 2) Wells A4-A11

 https://doi.org/10.3390/v17010092

contain drug B at 2x concentration that are serially diluted (1:2) from top to bottom (A toward H row). **Plate C:** the wells contain equal volumes from the corresponding wells in Plates A and B. Samples are mixed thoroughly before transferring them into the corresponding wells of the plate containing infected cells.

Table S1. Dose response synergistic score for IMP-1088 and MMF along with their cell viabilities for the corresponding doses.

IMP-1088 (µM)	MMF (µM)	ZIP_fit	ZIP_ref	ZIP_synergy	HSA_ref	HSA_synergy	Loewe_ref	Loewe_synergy	Loewe_ci	Bliss_ref	Bliss_synergy	% Cell viability	Std Cell viability
0.0078125	0.625	97.98	31.64	66.34	25.15	74.97	51.41	48.71	0.38	32.70	67.42	96.67	5.97
0.0078125	0.3125	75.89	10.58	65.31	10.13	68.86	23.32	55.67	0.41	12.44	66.56	96.93	6.12
0.015625	0.625	99.16	42.14	57.02	25.79	73.50	66.41	32.88	0.45	42.37	56.92	93.61	6.51
0.015625	0.3125	76.58	24.31	52.26	23.00	52.54	42.47	33.07	0.57	24.99	50.55	94.82	6.12
0.03125	0.625	101.25	62.28	38.97	49.10	50.99	81.84	18.25	0.51	61.89	38.20	93.75	3.5
0.0078125	0.15625	42.14	8.88	33.27	10.13	37.74	14.28	33.60	0.46	11.03	36.84	101.43	6.97
0.03125	0.3125	78.33	50.66	27.66	49.10	26.90	67.05	8.95	0.83	50.40	25.60	95.55	3.11
0.015625	0.15625	41.91	22.87	19.04	23.00	20.25	31.51	11.74	0.78	23.77	19.48	100.16	6.73
0.0625	0.625	101.22	83.96	17.26	76.32	23.69	93.65	6.36	0.69	82.27	17.74	94.1	1.94
0.0078125	1.25	102.83	88.62	14.21	87.72	15.49	91.63	11.58	0.49	88.96	14.26	94.6	6.23
0.015625	1.25	102.97	90.37	12.60	87.72	15.28	94.29	8.71	0.54	90.54	12.45	91.62	6.54
0.03125	1.25	103.23	93.72	9.51	87.72	15.77	97.48	6.00	0.49	93.75	9.74	91.44	3.87

Table S2. Prediction of myristoylated proteins in MPXV or VACV shows scores, predicted targeted sequence for myristoylation and visual depiction using both Myristoylator and MYR predictor tools.

						Myristoylator tool score		MYR prediciton server	
	Product	Virus	Description	Protein sequence	Protein ID	Positive	Negative	Sequence of the predicted myristoylation signal	Prediction Figure
1	MPXVgp079	MPXV	Entry/fusion complex component, myristylprotein (Cop-G9R) G10R myristylated protein similar to Vaccinia virus strain Copenhagen G9R	MGGGVSVELPKRDPPPGVPTDEMLLNVDKMHDVIAPAKLLEYV HIGPLTKDKEDKVKKRYPEFRLVNTGPGGLSALLRQSYNGTAPN CCRTFNRTHYWKKDGKISDKYEEGAVLESCWPDVHDTGKCDV DLFDWCQGDTFDINICHQWIGSAFNRSDRTVEGRQSLINLYNKM QRLCSKDASVPICELFLHHLRAHNTEDSKEMIDYILRQQSADFKQ KYMRCSYPTRDKLEESLKYAEPRECWDPECSNANVNFLLTRNY NNLGLCNIVRCNTSVNNLQMDKTSSLRLSCGLSNSDRFSTVPVN RAKVVQHNIKHSFDLKLHLISLLSLLVIWILIVAI*	URK20516.1	0.975045	0.0248832	GGGVSVELPKRDPPPGV	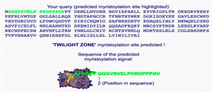
2	MPXVgp080	MPXV	IMV membrane protein (Cop-L1R) M1R myristylated IMV surface membrane protein similar to Vaccinia virus strain Copenhagen L1R	MGAAASIQTTVNTLSERISSKLEQEANASAQTKCDIEIGNFYIRQN HGCNITVKNMCSADADAQLDAVLSAATETYSGLTPEQKAYVPA MFTAALNIQTSVNTVVRDFENYVKQTCNSSAVVDNKLKIQNVIIDE CYGAPGSPTNLEFINTGSSKGNCAIKALMQLTTKATTQIAPRQVA GTGVQFYMIVIGVIILAALFMYYAKRMLFTSTNDKIKLILANKENVH WTTYMDTFFRTSPMIIATTDIQN*	URK20517.1	0.975005	0.0245614	GAAASIQTT VNTLSERI	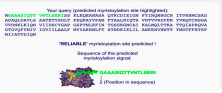
3	MPXVgp127	MPXV	A17L Myristylated protein, essential for entry/fusion (Cop-A16L) similar to Vaccinia virus strain Copenhagen A16L soluble myristylated protein	MGAAVTLNRIKIETGIADIRDKYMVLDFNYPEYNRAVRFAEESYM YYYETSPGEIKPKFCLIDGMSIDHCSSFIVPEFAKQYVLIHGEPCSS FKFRPGSLIYYQNEVTPEYIKDLKYATDYIASGQRCHFIKKDYLLG DSDSVAKCCSKTNTKHCPKIFNNNYKTEHCDDFMTGFCRNDPG NPNCLEWLRVKRKPAMSTYSDICSKHMDARYCSEFIRIIRPDYFT FGDTALYVFCNDHKGNRNCWCANYPKSNSGDKYLGPRVCWLH ECTDESRDRKWLYYNQDVQRTRCKYVGCTINVNSLALKNSQAE LTSNCTRTTSAVGDVHPGEPVVNDKIKLPTWLGASITLVVISVIFYF ISIYSRPKIKTNDINVRRR*	URK20565.1	0.516283	0.481462	GAAVTLNRI KIETGIAD	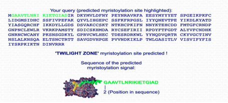
4	MPXVgp039	MPXV	C13L Hypothetical protein (Cop-F7L) similar to Vaccinia virus strain Copenhagen F7L	MGSCCGRFCDAKNKKEDVEDGREGCCDYKNLNDLDESEARVE FGPLYMINEEKSDINTLDIKRRYRHTIESVYF*	URK20474.1	0.0491496	0.950526	GSCCGRFCD AKNKKEDV	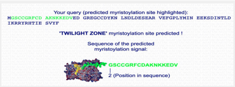
5	putative E7R	VACV		MGTAATIQTPTKLMNKENAEMILEKIVDHIVMYISDESSDSENNPE YIDFRNRYEDYRSLIIKSDHEFVKLCKNHAEKSSPETQQMIIKHIY EQYLIPVSEVLLKPIMSMGDIITYNGCKDNEWMLEQLSTLNFNNLR TWNSCSIGNVTRLFYTFFSYLMKDKLNI*	WDR17191.1	0.977976	0.0218719	GTAATIQTP TKLMNKEN	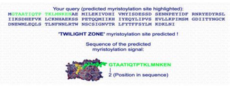
